# Comparing Large Language Models and Traditional Machine Translation Tools for Translating Medical Consultation Summaries: Quantitative Pilot Feasibility Study

**DOI:** 10.2196/85169

**Published:** 2026-04-13

**Authors:** Andy Li, Wei Zhou, Rashina Hoda, Chris Bain, Peter Poon

**Affiliations:** 1 Faculty of Information Technology, Monash University, Room 221, Clayton Campus, 20 Exhibition Walk, Clayton, 3800, Australia, +61 3 9902 9970; 2 Faculty of Medicine, Nursing and Health Sciences, Monash University, Wellington Rd, Clayton VIC 3800, Monash, Australia, 61 (03) 9905 4000; 3 Supportive and Palliative Care Unit, Monash Health, Monash, Australia

**Keywords:** feasibility study, pilot evaluation, translation, large language models, machine translation, consultation summary, responsible artificial intelligence, responsible AI, artificial intelligence, AI

## Abstract

**Background:**

Translation of medical consultation summaries is essential for equitable health care communication in culturally and linguistically diverse populations. While machine translation (MT) tools and large language models (LLMs) are widely accessible, their feasibility and safety for health care contexts remain underexplored.

**Objective:**

This pilot study investigates the feasibility and limitations of using LLMs and traditional MT tools to translate medical consultation summaries from English into the most common languages other than English spoken in Australia—Arabic, Chinese (simplified written form), and Vietnamese.

**Methods:**

Two simulated summaries—a simple patient-facing summary and a complex clinician-oriented interprofessional letter—were translated using 3 LLMs (GPT-4o, Llama-3.1, and Gemma-2) and 3 MT tools (Google Translate, Microsoft Bing Translator, and DeepL). Translations were benchmarked against professional third-party interpreter translations using Bilingual Evaluation Understudy, Character-level F-score, and Metric for Evaluation of Translation with Explicit Ordering metrics.

**Results:**

The translation performance varied across languages, tools, and summary complexity when assessed using automatic evaluation metrics. Traditional MT tools outperformed LLMs on surface-level metrics, while LLMs showed relative strengths in semantic similarity for Vietnamese and Chinese. Arabic translations improved with complex input, suggesting morphological advantages. The metric-based evaluation highlighted feasibility but also risks, particularly in Chinese clinical contexts.

**Conclusions:**

This pilot study provides formative evidence of opportunities and limitations in applying artificial intelligence translation for health care communication. Findings underscore the importance of human oversight; domain-specific evaluation metrics; and further formative and clinical research to guide the safe, equitable use of artificial intelligence translation tools.

## Introduction

Machine translation (MT) has seen rapid evolution in recent years, particularly with the advent of large language models (LLMs). Traditional neural MT tools, such as Google Translate [1], Microsoft Bing Translator [2], and DeepL [3], have been widely used in general and domain-specific applications. These tools rely heavily on sequence-to-sequence architectures and large-scale parallel corpora, showing strong performance for high-resource language pairs with abundant linguistic data. However, the effectiveness of MT is often constrained in domains that demand specialized vocabulary and contextual precision, such as medical communication [4]. While studies on digital translation in clinical care have reported improvements in communication quality and efficiency, they also highlighted limitations of MT in accuracy and further noted that direct, word-for-word translation, without accounting for cultural and contextual nuances, may lead to patient misunderstanding or even emotional distress [5,6].

In contrast, LLMs such as GPT [7], Gemma [8], and Llama [9] have emerged as versatile alternatives capable of performing a wide range of natural language tasks [10-16], including language translations [17,18]. These models leverage extensive pretraining on diverse corpora, enabling them to capture broader contextual relationships and discourse-level semantics across languages. Previous studies have shown that LLMs can outperform traditional MT tools in document-level linguistic translation by better preserving coherence and semantic intent [17]. Moreover, recent work has shown that LLMs can act as effective quality estimators for translation output, even without explicit references [18].

Despite these advances, the application of LLMs in medical translation remains underexplored and presents unique challenges [19,20]. Medical texts require not only accurate translation of terminology and clinical concepts but also contextual sensitivity, as even small errors can lead to harm [4]. Terminological consistency, expansion of abbreviations, and the handling of multilingual clinical guidelines add layers of complexity [21]. These challenges are heightened in multilingual health care settings such as Australia, where languages such as Arabic, Chinese, and Vietnamese are widely spoken but often underrepresented in medical translation tools [22]. The accuracy and safety of using general-purpose LLMs in such contexts remain largely untested.

This pilot feasibility study builds on existing research by providing an early-stage, empirical comparison of LLMs and traditional MT tools in translating palliative care consultation summaries—a use case that is both medically sensitive and linguistically nuanced [23]. Our study focuses on 3 of the most spoken languages other than English in Australia—Arabic, Chinese, and Vietnamese—each posing different linguistic and morphological challenges [22]. While Vietnamese, like many other languages, is relatively undersupported in traditional MT tools (eg, not supported by DeepL), LLMs have the potential to fill these gaps through flexible, prompt-based translation.

We designed 2 types of simulated summaries in English to reflect real-world use cases: a simple summary for patients, written in lay language and a complex, clinician-targeted version featuring domain-specific jargon, abbreviations, and nuanced medical reasoning. Translations were generated using default prompt settings for each LLM and default web versions of each MT tool and evaluated against professional third-party medical interpreter translations. Automatic evaluation metrics—Bilingual Evaluation Understudy (BLEU) [24], Character-level F-score (CHR-F) [25], and Metric for Evaluation of Translation with Explicit Ordering (METEOR) [26]—were used to capture surface-level overlap, morphological robustness, and semantic similarity, respectively [27]. This multifaceted analysis allowed us to assess the models not only in terms of fidelity to reference but also their ability to generalize across languages and document complexity.

By comparing performance across tool types, summary complexity, and languages, this formative study provides insights into the current feasibility and limitations of LLM-based translation for medical applications. Our findings highlight language-specific challenges, the shortcomings of current evaluation metrics for clinical accuracy, and the implications of deploying AI translation tools in health care communication. Importantly, translation errors in clinical contexts can compromise patient safety, reduce provider efficiency, and hinder equitable access to care [28].

This study is intentionally designed as a *pilot feasibility investigation*. Its goal is not to benchmark optimal system performance or establish clinical readiness but to explore practical risks, limitations, and methodological challenges of using LLMs and MT tools for medical translation and to inform the design of future, clinically grounded evaluation studies.

## Methods

### Overview

This pilot feasibility study presents an early-stage comparison between popular LLMs and traditional MT tools in translating medical documents. We selected the latest and most capable variants of 3 state-of-the-art LLMs: GPT-4o (OpenAI), Gemma-2-27B (Google), and Llama-3.1-405B (Meta). For traditional MT tools, we selected 3 widely used services: Google Translate, Microsoft Bing Translator, and DeepL. LLMs have demonstrated strong capabilities in general-purpose translation, and commercial translation products based on LLMs have already been introduced into the market. However, the extent to which LLMs can be reliably and safely used for translating medical texts, especially in safety-critical areas such as digital health, remains largely unexplored. This study aims to investigate this gap by evaluating translation quality across both LLMs and traditional MT tools. Our design was intentionally small scale and exploratory, with the goal of generating formative evidence to inform larger clinical studies.

### Ethical Considerations

This study used entirely fictitious medical consultation summaries that were constructed by an experienced palliative care clinician and did not contain any real patient information, personally identifiable data, or clinical records. As a result, formal human research ethics approval was not required.

### Original Summaries in English

To simulate realistic clinical use cases, we created 2 types of fictitious consultation summaries in English. The first is a short and relatively simple summary with minimal medical terminology intended for patient-facing medical documents. It summarizes a simulated palliative care consultation of a patient with progressive dementia who recently recovered from a urinary tract infection. It captures the necessary components of a patient-facing summary, including medical history, recent care updates, and future recommendations. The second summary is longer and more complex, featuring extensive medical jargon, including abbreviations and acronyms, typically used for communication purposes between clinicians (eg, correspondence letter between the specialist and family physicians) or toward health care provider documents such as discharge summaries. These summaries were designed to reflect common clinical communication scenarios while ensuring no real patient data were used. The complex summary described a patient with advanced acute myeloid leukemia and treatment complications, requiring specialized care.

It is important to emphasize that the summaries were entirely fictitious and meticulously constructed by a qualified palliative care expert with over 25 years of clinical experience. No personally identifiable information or sensitive patient data were included. This ensured compliance with ethical standards and minimized risks to participants, in line with formative study expectations.

### Selected Languages

The target languages for translation were Arabic, Chinese (simplified), and Vietnamese, which were chosen because they are the 3 most commonly spoken languages other than English in Australia, as reported by the Australian Bureau of Statistics [22]. Due to DeepL not supporting Vietnamese at the time of the study, its outputs for that language were not included in our analysis. The language set was chosen to reflect the culturally and linguistically diverse populations most relevant to the Australian health care system.

### Reference Translations

The reference translations for both summaries were generated by a professional third-party translation service with extensive experience in translating medical documents. The service is certified under ISO 17100, an international standard that establishes requirements to ensure the quality of translation services. All translators involved were accredited by the National Accreditation Authority for Translators and Interpreters. Each translation underwent a rigorous quality assurance process, which included a review by an independent translator (ie, a linguist not involved in the original translation task or providing verification) to identify typographical errors, formatting inconsistencies, untranslated or mistranslated content, and placeholder artifacts that may arise during document handling or after editing.

To ensure that reference translations were reflective of industry practice and standards, we engaged accredited translation services. However, it is important to note that the contemporary professional translation workflow commonly incorporates machine-generated drafts as a starting point for human postediting. There are no regulations that prohibit the use of machine-generated translations, and ISO 17100 also incorporates guidelines for using machine-generated translations as preliminary drafts. Consequently, while every effort was made to ensure the quality and accuracy of the translations, obtaining translations that are purely human generated from first principles is increasingly difficult in modern professional settings. While the translation provider indicated that the Chinese and Vietnamese translations were produced entirely by human translators, the first draft of the Arabic translation was generated with the assistance of artificial intelligence (AI)–based translation tools and later reviewed by the human translator. This further underscores the increasingly widespread adoption and normalization of generative AI technologies to assist professional translation services.

### Generated Translations

For each summary, 2 sets of translations were generated as shown in the translation task matrix (Table 1). The first set consisted of 3 LLM-generated translations. This experiment aimed to mimic laypeople using LLMs as a translation service; therefore, the basic web platform was used instead of the developer’s application programming interface, which means alll parameters (including system prompt) were set as the model’s default values. All LLM-generated translations also used the same basic prompt: “Can you translate this document into Arabic/Chinese/Vietnamese, make sure no information is lost. ‘*DOCUMENT*’,” where *DOCUMENT* is the original summary. All LLM experiments in this study were conducted using a *zero-shot setting*, without any examples, prompt tuning, or system-level customization. The single, minimal prompt was intentionally chosen to reflect typical lay-user (eg, patients) behavior, rather than to evaluate the maximum achievable capability of each model. No terminology constraints, abbreviation handling rules, unit normalization, or do-not-translate rules were provided. Therefore, the results should be interpreted as reflecting realistic, nonexpert use, not optimized or clinician-engineered performance.

**Table 1.** Translation task matrix (summary type×language×system).^a^

Summary type and language	GPT	Gemma	Llama	Google Translate	Microsoft Bing	DeepL
Simple						
Arabic	✓	✓	✓	✓	✓	✓
Chinese	✓	✓	✓	✓	✓	✓
Vietnamese	✓	✓	✓	✓	✓	
Complex						
Arabic	✓	✓	✓	✓	✓	✓
Chinese	✓	✓	✓	✓	✓	✓
Vietnamese	✓	✓	✓	✓	✓	

a DeepL did not support Vietnamese translation at the time of this study. A total of 34 translations were produced across 36 combinations.

The second set of translations was produced using traditional MT tools—Google Translate, Microsoft Bing Translator, and DeepL—by inputting the same English summaries into their publicly accessible web interfaces. As with the LLMs, no customization or advanced application programming interface use was applied, ensuring that the outputs reflect a typical user experience. This approach reflects feasibility testing rather than optimized performance.

### Statistical Analysis

We used 3 widely used automatic metrics from the MT domain to obtain a comprehensive assessment for each generated summary. These were BLEU, CHR-F, and METEOR. Although originally developed for MT, these metrics remain widely applied to LLM outputs and were used here to provide indicative, rather than definitive, measures of quality [19,29]. Currently, there are no standardized evaluation metrics specifically designed for LLM-generated translations, although recent studies suggest LLMs themselves may serve as quality estimators [18]. A brief description of each metric used and its limitations are listed in Table 2. The values of each metric range from 0 to 1, where 1 indicates an identical match between the reference translation and generated translation, while 0 indicates no overlap at all.

**Table 2.** Description and limitations of each statistical metric used in the evaluation.

Metric	Description	Limitation
BLEU (Bilingual Evaluation Understudy)	Measures the word level of overlapping between candidate and reference translations. It is the most common metric in machine translation.	Lacks acceptance of synonyms, paraphrasing, and overall semantic meaning.
CHR-F (Character-level F-score)	Measures the character level of overlapping between candidate and reference translations, making it particularly effective for languages with complex morphology or segmentation issues.	Lacks acceptance of synonyms, paraphrasing, and overall semantic meaning.
METEOR (Metric for Evaluation of Translation with Explicit Ordering)	Aligns words between candidate and reference translations, incorporating synonyms, stemming, and paraphrasing, offering a balance between surface and semantic matching, and is also sensitive to syntax.	Struggles with complex semantics or meanings that require deep understanding.

Specifically, BLEU measures the *surface similarity at the word level compared to the reference translation*. A high score in BLEU in the health setting means the medical terminology and linguistics for symptoms or diagnosis are captured accurately. CHR-F measures the *surface similarity at the character level compared to the reference translation*, which handles morphological variation better than BLEU. A high CHR-F score means that the generated translation is capturing medical jargon, especially abbreviations, better. METEOR measures *similarity at the semantic level*. A high METEOR score indicates that even when a medical condition or advice is expressed differently, the core semantic content is still preserved.

## Results

### Overview

Table 3 presents a comparative evaluation of translation quality for 2 English medical consultation summaries—simple (layperson-friendly) and complex (clinician-oriented)— translated into Arabic, Chinese, and Vietnamese using 3 LLMs (GPT-4o, Llama-3.1, and Gemma-2) and 3 traditional MT tools (Google Translate, Microsoft Bing Translator, and DeepL). Translation outputs were scored using 3 established automatic evaluation metrics: BLEU (for surface-level n-gram overlap), CHR-F (for character-level fidelity), and METEOR (for semantic similarity and paraphrasing tolerance).

**Table 3.** Translation performance comparison across 3 automatic evaluation metrics—Bilingual Evaluation Understudy (BLEU), Character-level F-score (CHR-F), and Metric for Evaluation of Translation with Explicit Ordering (METEOR) —for 3 LLMs (GPT-4o, Llama-3.1, and Gemma-2) and 3 traditional MT tools (Google Translate, Microsoft Bing Translator, and DeepL).

	GPT	Llama	Gemma	Microsoft Bing Translator	Google Translate	DeepL
Arabic						
Simple						
BLEU	0.6536	0.5947	0.6051	0.6405	0.6528	0.6523
CHR-F	0.5064	0.4562	0.4693	0.5078	0.5151	0.5156
METEOR	0.3166	0.2568	0.2810	0.3289	0.3399	0.3048
Complex						
BLEU	0.6300	0.6029	0.6006	0.6149	0.6787	0.6692
CHR-F	0.4957	0.4726	0.4768	0.5005	0.5907	0.5423
METEOR	0.3343	0.3575	0.3682	0.4012	0.4988	0.4171
Chinese						
Simple						
BLEU	0.4266	0.4164	0.5227	0.5018	0.4358	0.4322
CHR-F	0.4165	0.3910	0.4392	0.4193	0.3709	0.3844
METEOR	0.4698	0.5033	0.5678	0.5169	0.4564	0.5097
Complex						
BLEU	0.2662	0.2422	0.2466	0.2393	0.2596	0.2603
CHR-F	0.2871	0.2387	0.2576	0.2427	0.2729	0.2526
METEOR	0.3055	0.3347	0.3394	0.3294	0.3349	0.3331
Vietnamese						
Simple						
BLEU	0.7188	0.7517	0.7458	0.7464	0.7719	—^a^
CHR-F	0.5956	0.5979	0.5957	0.5864	0.6184	—
METEOR	0.5566	0.5894	0.5489	0.5373	0.5908	—
Complex						
BLEU	0.6857	0.7441	0.7118	0.7059	0.7325	—
CHR-F	0.5494	0.6055	0.6130	0.5862	0.6252	—
METEOR	0.4652	0.4787	0.5399	0.5222	0.5744	—

a Not available.

### Simple vs Complex Summary Translation Performance

We observed a consistent pattern across the Chinese and Vietnamese languages, where simple summaries—written in layperson language with minimal technical jargon—achieved higher scores across most metrics compared with complex summaries. This trend was less pronounced or even reversed in Arabic. For example, in Vietnamese, BLEU scores for the simple summary were high across all models, with Google Translate achieving the highest score of 0.7719, followed closely by Llama (0.7517) and MS (0.7464). CHR-F and METEOR scores also reflected strong alignment, with Google Translate again leading in METEOR score (0.5908).

However, for the complex summary, BLEU scores dipped across all models (eg, GPT declined from 0.7188 to 0.6857), although the degradation for Vietnamese was relatively minor, suggesting good resilience for Vietnamese translation. A dramatic drop was observed in Chinese performance. For instance, Gemma’s BLEU score fell from 0.5227 (simple) to 0.2466 (complex). CHR-F and METEOR scores showed a similar drop, with CHR-F scores dropping from 0.4392 to 0.2576 and METEOR scores dropping from 0.5678 to 0.3394. This suggests a struggle in translating complex, technical content due to syntactic and terminological challenges.

In Arabic, an interesting reverse trend emerged. For instance, Google Translate’s BLEU score increased from 0.6528 (simple) to 0.6787 (complex). The METEOR score also jumped significantly, from 0.3399 to 0.4988. This may be due to Arabic’s rich morphology, where longer, more context-rich sentences provide better clues for disambiguation and grammatical accuracy.

### LLMs vs Traditional MT Tools

Across all languages, traditional MT tools (Google Translate and Microsoft Bing Translator) generally outperformed LLMs such as GPT-4o, Llama, and Gemma on standard metrics, particularly for complex summaries.

For example, in Arabic complex summaries, Google Translate achieved a BLEU score of 0.6787 and a METEOR score of 0.4988, outperforming GPT (BLEU: 0.6300; METEOR: 0.3343), Llama, and Gemma. Similar trends were seen in CHR-F, where Google Translate scored 0.5907, the highest among all systems.

This reflects the focus of traditional MT tools on token-level alignment and training objectives that favor metrics such as BLEU and CHR-F, rewarding close n-gram matches. On the other hand, LLMs often prioritize fluency and coherence, producing paraphrased outputs that, while semantically accurate, may score lower due to structural differences.

However, Llama outperformed GPT-4o in Vietnamese and Chinese METEOR scores, and Gemma did the same except for Vietnamese simple summaries. For example, in Vietnamese simple summaries, Llama outperformed GPT with a METEOR score of 0.5894 vs 0.5566, suggesting that specific LLMs may better capture semantic nuances in certain linguistic contexts.

### Language-Specific Performance Trends

The 3 target languages demonstrated distinct translation characteristics in response to summary complexity and model type:

- Arabic: translation quality improved with complex summaries across most models. For instance, GPT’s CHR-F score went from 0.5064 (simple) to 0.4957 (complex)—a small drop, but METEOR scores rose from 0.3166 to 0.3343 and other models (such as Llama and Gemma) saw larger METEOR score gains. This supports the hypothesis that longer, more redundant input helps Arabic models resolve morphological ambiguities.
- Chinese: performance dropped sharply from simple to complex summaries. BLEU scores for Gemma fell from 0.5227 to 0.2466 and METEOR fell from 0.5678 to 0.3394. This suggests transliteration issues, syntactic mismatches, and rare term handling present challenges in Chinese clinical text translation. Similar challenges in Chinese medical translation have been reported in previous studies, where syntactic ambiguity, segmentation issues, and limited domain-specific parallel corpora reduce translation accuracy [21].
- Vietnamese: scores declined modestly from simple to complex, showing the least performance degradation. For example, Llama’s BLEU score only dropped from 0.7517 to 0.7441 and CHR-F score actually increased from 0.5979 to 0.6055. This suggests that Vietnamese translation is robust, possibly due to better multilingual representation in modern models.

## Discussion

### Principal Findings

This pilot feasibility study compared LLMs and traditional MT tools for translating medical consultation summaries into Arabic, Chinese, and Vietnamese. Translation performance varied substantially by language, document complexity, and system type.

Overall, traditional MT tools achieved higher scores on surface-level metrics such as BLEU and CHR-F, particularly for complex, clinician-oriented summaries, reflecting optimization for lexical fidelity. In contrast, LLMs demonstrated relative strengths in semantic similarity in selected language-summary combinations, as reflected by METEOR scores, particularly for Vietnamese and Chinese simple summaries. These patterns suggest that while traditional MT tools may better preserve surface form, LLMs may capture broader semantic intent in some contexts.

Language-specific trends further illustrate the complexity of multilingual medical translation. Chinese translations showed a marked decline in performance for complex summaries, highlighting challenges related to segmentation and specialized terminology. Vietnamese translations were comparatively robust across summary complexity, while Arabic translations improved for more complex input, potentially due to increased contextual redundancy supporting disambiguation in morphologically rich language structures. Importantly, metric-based performance alone does not indicate clinical safety or appropriateness for health care use.

### Implications

These findings should be interpreted in light of how translation quality is evaluated and how AI translation tools are used in real-world health care settings. Differences observed between systems reflect not only model capability but also the interaction between evaluation metrics, linguistic structure, and user behavior.

Automatic translation metrics such as BLEU and CHR-F prioritize surface-level similarity and therefore favor systems optimized for n-gram alignment. METEOR partially accounts for paraphrasing but remains unable to capture clinical salience, pragmatic intent, or contextual appropriateness. As a result, metric differences should not be interpreted as indicators of clinical safety. In health care communication, mistranslating a medication name or dosage carries far greater risk than stylistic variation, yet current metrics penalize both equally [30,31]. This reflects a broader challenge in evaluating AI-generated medical translations.

Differences between LLMs and traditional MT tools further illustrate this issue. Traditional MT systems generally achieved higher surface-level scores, while LLMs often produced more fluent and paraphrased outputs that diverged structurally from professional reference translations. Reliance on automatic metrics alone may therefore underestimate communicative strengths while simultaneously obscuring clinically meaningful errors that require expert judgment to identify.

The observed language-specific patterns underscore the need for language-aware evaluation approaches. Performance differences across Arabic, Chinese, and Vietnamese suggest that linguistic structure, morphology, and contextual density influence how effectively models disambiguate clinical meaning. These findings caution against assuming uniform translation performance across languages in health care contexts.

Finally, the results must be interpreted in the context of real-world use. The workflows evaluated in this study intentionally reflect nonexpert use, where patients or carers rely on publicly accessible tools without structured prompts, terminology constraints, or professional oversight. Translation quality in LLMs is known to be prompt sensitive [32,33], and professional translation workflows increasingly involve human postediting of machine-generated drafts. Accordingly, the findings represent feasibility and risk under typical lay-user conditions rather than upper-bound system capability.

### Limitations and Future Directions

This study has several limitations that define its methodological scope and directly inform future research priorities.

First, the experimental design reflects lay-user translation behavior rather than professional or clinical workflows. We used a single minimal prompt for LLMs and default web interfaces for MT tools, without terminology constraints, glossary enforcement, abbreviation expansion, or structured output formats. While such safeguards are standard in clinical translation services, they are rarely applied by nonexpert users. Future work should systematically compare lay-user workflows with clinically realistic translation pipelines to distinguish model capability from misuse-related risk.

Second, no human expert adjudication was conducted. Without clinician or professional translator review, it is not possible to classify error types, assess severity, or determine potential impacts on patient understanding or clinical decision-making [28,30,31,34]. Future studies should incorporate structured human-in-the-loop evaluation to identify safety-critical errors.

Third, the study is deliberately small in scale, using 2 simulated consultation summaries representing limited clinical contexts. This precludes generalization across specialties, documentation styles, or real-world variability. Larger multidomain datasets are required to assess generalizability.

Fourth, statistical reporting is limited to point estimates of automatic metrics. CIs, uncertainty estimation, and robustness analyses were not performed due to the small sample size. Future evaluations should adopt statistically robust analysis frameworks while avoiding overinterpretation of metric differences. While some studies indicate that LLMs themselves can be used as evaluators to grade the quality of translation, the research is still at a very early stage and lacks a comprehensive understanding of the limitations [35].

Finally, existing automatic metrics are poorly aligned with clinical risk and cannot distinguish stylistic variation from safety-critical errors. There is a clear need for translation-specific clinical evaluation frameworks that integrate human judgment and explicitly assess dimensions such as accuracy of critical entities, hallucination rate, trustworthiness, and interpretability in multilingual health care contexts [35-37].

Together, these limitations and future directions underscore that the primary contribution of this study is not to establish clinical readiness but to clarify key risks, evaluation gaps, and methodological challenges that must be addressed before AI-assisted medical translation can be responsibly used in health care communication.

### Conclusion

This pilot feasibility study provides an early, structured comparison of LLMs and traditional MT tools for translating medical consultation summaries into Arabic, Chinese, and Vietnamese within a palliative care context. The findings highlight that while both system types can produce translations that appear usable under automatic evaluation metrics, performance varies substantially by language, document complexity, and evaluation method. Importantly, metric-based similarity does not equate to clinical safety, and unstructured use of AI translation tools—particularly by nonexpert users—poses tangible risks in health care communication.

This pilot feasibility study does not assess clinical safety or readiness for deployment. Instead, it clarifies key methodological challenges and evaluation gaps that must be addressed before AI-assisted medical translation can be responsibly integrated into health care practice. Human oversight and clinically grounded evaluation remain essential.

## Abbreviations

AI: artificial intelligence
BLEU: Bilingual Evaluation Understudy
CHR-F: Character-level F-score
LLM: large language model
METEOR: Metric for Evaluation of Translation with Explicit Ordering
MT: machine translation
